# Association Between DLCO and Echocardiographic Right Ventricular–Pulmonary Arterial Coupling in Idiopathic Pulmonary Fibrosis

**DOI:** 10.3390/jcm15176833

**Published:** 2026-09-03

**Authors:** Francesca Coppi, Susan Darroudi, Giulia Renda, Alessandra Lodeserto, Arianna Maini, Damiano De Cesare, Youssef Othman, Martina Mercati, Dalia Caleffi, Filippo Gozzi, Francesco Marangi, Dario Andrisani, Federica Andolfi, Alessio Baccarani, Daniela Aschieri, Anna Vittoria Mattioli, Francesco Fedele, Enrico Clini, Stefania Cerri, Gianluca Pagnoni

**Affiliations:** 1 Department of Medical and Surgical Sciences for Children and Adults, University of Modena and Reggio Emilia, Via del Pozzo 71, 41124 Modena, Italy; francesca.coppi@unimore.it (F.C.); darroudis921@gmail.com (S.D.); ariannamm@live.it (A.M.); decesaredamiano@gmail.com (D.D.C.); 287583@studenti.unimore.it (Y.O.); 266588@studenti.unimore.it (M.M.);; 2 National Institute for Cardiovascular Research (INRC), Via Irnerio 48, 40126 Bologna, Italy; annavittoria.mattioli@unibo.it (A.V.M.); francesco.fedele@uniroma1.it (F.F.); 3 Department of Neuroscience, Imaging and Clinical Sciences, G. d’Annunzio University of Chieti-Pescara, 66100 Chieti, Italy; grenda@unich.it; 4 Pneumology Unit, Department of Medical and Surgical Sciences, University of Modena and Reggio Emilia, 41121 Modena, Italy; 342753@studenti.unimore.it; 5 Cardiology Division, Azienda Ospedaliera Universitaria di Modena, 41124 Modena, Italy; dalia.caleffi@unimore.it; 6 Centre for Rare Lung Diseases, Pneumology Unit, Department of Medical and Surgical Sciences, ERN-Lung Core Network ILD, University Hospital of Modena—Policlinico, University of Modena and Reggio Emilia, 41121 Modena, Italy; gozzi.filippo@aou.mo.it (F.G.); andrisani.dario@aou.mo.it (D.A.); federicaandolfi95@gmail.com (F.A.); enrico.clini@unimore.it (E.C.); 7 Division of Plastic and Reconstructive Surgery, Department of Medical and Surgical Sciences, University Hospital of Modena—Policlinico, University of Modena and Reggio Emilia, 41124 Modena, Italy; alessio.baccarani@unimore.it; 8 Cardiology Unit of Emergency Department, Guglielmo da Saliceto Hospital, 29121 Piacenza, Italy; d.aschieri@ausl.pc.it (D.A.); dr.gianlucapagnoni@gmail.com (G.P.); 9 Department of Quality, Life University of Bologna—Alma Mater Studiorum, 40126 Bologna, Italy; 10 Emeritus of Cardiology, Sapienza University of Rome, 00189 Rome, Italy

**Keywords:** idiopathic pulmonary fibrosis, pulmonary diffusing capacity, echocardiography, ventricular function, right, hypertension, pulmonary

## Abstract

**Background:** Idiopathic pulmonary fibrosis (IPF) is frequently complicated by pulmonary vascular involvement and right ventricular (RV) dysfunction, both of which adversely affect prognosis. The relationship between impaired gas exchange and echocardiographic indices of RV function, particularly the TAPSE/PAPs (tricuspid annular plane systolic excursion/systolic pulmonary artery pressure) ratio as a marker of RV–pulmonary arterial (RV–PA) coupling, has not been fully characterized. **Methods:** We retrospectively evaluated 198 consecutive patients with IPF assessed at a single tertiary referral center between 2020 and 2026; 188 with complete pulmonary function testing were included in the main analyses. The primary exposure was gas exchange, measured by single-breath DLCO% (diffusing capacity of the lung for carbon monoxide) and stratified into four categories (Normal ≥ 80%, n = 21; Mild 60–79%, n = 56; Moderate 40–59%, n = 70; Severe < 40%, n = 41). The main outcome measures were echocardiographic indices of RV function—TAPSE, systolic pulmonary artery pressure (PAPs), and the TAPSE/PAPs ratio. Associations were assessed using Pearson’s correlation, one-way ANOVA, and multivariable linear regression adjusted for age, sex, and smoking status, with the Mild group as the reference for categorical analyses. **Results:** Lower DLCO values were independently associated with higher PAPs (adjusted β = −0.24 mmHg per 1% DLCO, *p* = 0.004) and with a lower TAPSE/PAPs ratio (adjusted β = +0.005 per 1% DLCO, *p* = 0.040). Compared with the Mild reference group, patients with Severe DLCO reduction had markedly higher PAPs (+14.6 mmHg, *p* = 0.002) and a lower TAPSE/PAPs ratio (−0.37, *p* = 0.002), with attenuated findings in the Moderate category; the small Normal group did not differ significantly from the Mild one. TAPSE alone showed only a weak, non-significant trend (r = 0.195, *p* = 0.083). **Conclusions:** In patients with IPF, lower DLCO was independently associated with higher pulmonary artery pressure and, to a lesser extent, with reduced RV–PA coupling (a lower TAPSE/PAPs ratio), whereas RV longitudinal systolic function (TAPSE) appeared preserved. Among pulmonary function parameters, DLCO showed the strongest association with PAPs. In this exploratory, cross-sectional analysis, combined evaluation of DLCO and the TAPSE/PAPs ratio may help characterize right heart involvement in IPF; its value for pulmonary hypertension (PH) screening requires prospective confirmation.

## 1. Introduction

Idiopathic pulmonary fibrosis (IPF) is a chronic, progressive interstitial lung disease of unknown etiology, characterized by a relentless deposition of fibrotic tissue in the lung parenchyma and a consequent loss of respiratory function. The disease predominantly affects older adults and is associated with a poor prognosis, with a median survival of approximately 3–5 years from diagnosis when left untreated [1,2]. Patients typically present with progressive exertional dyspnea, a chronic non-productive cough, and a gradual decline in gas exchange that ultimately leads to chronic hypoxemia and a marked reduction in functional capacity and quality of life [3].

Although IPF is primarily considered a pulmonary disease, its progression is intimately linked to the cardiovascular system, particularly the right heart [4]. The fibrotic remodeling of the lung parenchyma and the associated pulmonary vascular involvement lead to an increase in pulmonary vascular resistance, which in turn imposes a progressive pressure overload on the right ventricle (RV). Because the RV is anatomically and physiologically designed as a low-pressure pump, it adapts poorly to sustained increases in afterload. Over time, this mismatch can result in RV dysfunction, uncoupling between the right ventricle and the pulmonary circulation, and ultimately overt right heart failure events that are all associated with poor clinical outcomes in patients with IPF [5].

Echocardiography is a non-invasive, widely available, and reproducible imaging modality that plays a central role in the assessment of the right heart in IPF. Among the parameters used to evaluate right ventricular performance, the tricuspid annular plane systolic excursion (TAPSE) provides a simple and robust estimate of RV longitudinal systolic function, while tissue Doppler imaging (S′ TDI) adds complementary information on myocardial velocity [6,7]. Recent reproducibility data comparing these two parameters further support their use in the standardized echocardiographic evaluation of RV function [8]. Systolic pulmonary artery pressure (PAPs), estimated from the tricuspid regurgitation jet, reflects the afterload imposed on the RV. More recently, the TAPSE/PAPs ratio has emerged as a non-invasive surrogate of right ventricular–pulmonary arterial (RV–PA) coupling, integrating contractile function and vascular load into a single, clinically meaningful index [9]. This ratio has been validated against the invasively measured ratio of right ventricular end-systolic to pulmonary arterial elastance, and a value below 0.31 mm/mmHg identifies impaired coupling and independently predicts mortality in pulmonary hypertension [10,11]. Current recommendations for the echocardiographic assessment of the right heart—including the 2022 ESC/ERS pulmonary hypertension guidelines and the recent American Society of Echocardiography recommendations—endorse an integrated evaluation of RV function together with afterload rather than reliance on any single parameter [7,8,12].

Pulmonary function tests (PFTs) are the cornerstone of functional evaluation in IPF [4]. Forced vital capacity (FVC) and total lung capacity (TLC) reflect the restrictive physiology typical of the disease, whereas the diffusing capacity for carbon monoxide (DLCO) integrates information on alveolar–capillary membrane integrity and pulmonary microvascular bed [13,14]. A reduction in DLCO out of proportion to the reduction in lung volumes has long been recognized as an indirect marker of pulmonary vascular involvement and has been associated with the development of pulmonary hypertension in several interstitial lung diseases. In this context, DLCO may represent an early and sensitive indicator of the pulmonary vascular remodeling that precedes the onset of overt RV dysfunction [15]. Because DLCO depends on the surface area and integrity of the alveolar–capillary interface, its reduction reflects not only fibrotic destruction of the parenchyma but also the progressive rarefaction of the pulmonary microvasculature—capillary loss, vascular pruning, intimal fibrosis, and medial hypertrophy—that characterizes the vascular remodeling of IPF [5,16]. This microvascular rarefaction increases pulmonary vascular resistance and the afterload imposed on the right ventricle, providing a mechanistic link between impaired gas exchange and deterioration of RV–PA coupling.

Despite growing interest in the cardiopulmonary interaction in IPF, the relationship between pulmonary function parameters and echocardiographic indices of RV function, including the novel RV–PA coupling ratio, has not been fully characterized. Most of the existing literature has focused either on the prognostic value of isolated echocardiographic parameters or on the presence of pulmonary hypertension in advanced disease, while a comprehensive, integrated evaluation of how progressive impairment of gas exchange and lung volumes translates into measurable changes in right heart structure and function remains limited. We focused on DLCO and echocardiography because both are simple, non-invasive, and widely available in routine IPF care: among pulmonary function parameters, DLCO is the most sensitive to pulmonary vascular involvement, whereas echocardiography provides reproducible, radiation-free assessment of right ventricular structure, function, and afterload without the need for right heart catheterization, together allowing for non-invasive cardiopulmonary phenotyping in everyday clinical practice. The aim of the present study was therefore to evaluate, in a cohort of patients with IPF, the association between pulmonary function, assessed by DLCO and FVC, and right ventricular function, assessed by echocardiographic parameters, specifically TAPSE, PAPs, and the TAPSE/PAPs ratio. We hypothesized that progressively lower DLCO, reflecting greater loss of the pulmonary microvascular bed, would be independently associated with higher PAPs and a lower TAPSE/PAPs ratio, that is, worse RV–PA coupling, whereas TAPSE alone would remain relatively preserved. The primary hypothesis concerned the association between DLCO and the TAPSE/PAPs ratio, with PAPs and TAPSE examined as secondary outcomes.

## 2. Materials and Methods

### 2.1. Study Design and Population

This was a retrospective observational study conducted at the Cardiology and Pneumology outpatient clinics of the University Hospital of Modena and Reggio Emilia, Italy. A total of 198 consecutive patients with a confirmed diagnosis of idiopathic pulmonary fibrosis (IPF) and available clinical follow-up between 2020 and 2026 were included. Of these, 188 patients had completed pulmonary function tests and were included in the main analyses. The diagnosis of IPF was established according to the 2018 ATS/ERS/JRS/ALAT clinical practice guidelines [17], based on a multidisciplinary evaluation integrating clinical, radiological (HRCT), and, when available, histological criteria [17]. Exclusion criteria were defined to minimize the influence of cardiac or pulmonary conditions other than IPF on the echocardiographic and functional parameters of interest. Specifically, patients were excluded if they presented with: (i) significant left-sided heart disease, including left ventricular ejection fraction <50%, moderate-to-severe left ventricular diastolic dysfunction, or known clinical heart failure; (ii) moderate-to-severe valvular heart disease (mitral or aortic stenosis or regurgitation greater than mild); (iii) congenital heart disease; (iv) known pulmonary hypertension of other etiologies (WHO Groups 1, 4, or 5 PH); (v) chronic thromboembolic pulmonary disease; (vi) significant chronic obstructive pulmonary disease (FEV1/FVC < 0.70 with FEV1 < 50% predicted) or other interstitial lung diseases overlapping with IPF; and (vii) incomplete clinical or instrumental records preventing the assessment of the main study variables.

### 2.2. Data Collection

Clinical, laboratory, and instrumental data were extracted from the patients’ electronic medical records and organized in a dedicated study database. For each patient, the following variables were collected: demographic and anthropometric data (age at diagnosis, sex, BMI), smoking history (never, former, current smoker), and main cardiovascular risk factors (arterial hypertension, dyslipidemia, diabetes mellitus). The HRCT pattern at diagnosis was recorded according to the 2018 ATS/ERS classification (definite UIP, probable UIP, indeterminate, alternative diagnosis), together with the presence of concomitant emphysema [17].

Laboratory parameters included complete blood count (hemoglobin, MCV, MCHC, RDW), renal function, and serum 25-hydroxy-vitamin D levels, when available.

### 2.3. Pulmonary Function Tests

Pulmonary function test data were retrospectively retrieved from existing clinical records. All tests had been performed during routine clinical assessment at the Pulmonology service of the University Hospital of Modena and Reggio Emilia using standardized equipment (Autobox DL 6200 (SensorMedics, Yorba Linda, CA, USA)), consistent with the contemporary ATS/ERS technical standards [17]. The following parameters, expressed as percentage of predicted values, were considered: forced vital capacity (FVC), forced expiratory volume in the first second (FEV1), total lung capacity (TLC), single-breath diffusing capacity for carbon monoxide (DLCO-Sb) and DLCO corrected for alveolar volume (DLCO/VA). For the purpose of the analyses, the DLCO-Sb % was used as the primary measure of gas exchange. Patients were stratified into four categories of DLCO severity according to standard clinical cut-offs: severe (<40%), moderate (40–59%), mild (60–79%), and normal (≥80%). For each patient, the pulmonary function test performed in closest temporal proximity to the echocardiographic examination was selected. Although the retrospective design did not allow the exact dates to be retrieved in every case, the interval between the two assessments ranged from a few days to a few weeks and was in all cases shorter than three months.

### 2.4. Echocardiographic Assessment

As part of routine clinical assessment, the majority of patients underwent transthoracic echocardiographic examination (Philips Healthcare, Amsterdam, The Netherlands), performed by experienced operators according to standard clinical practice and consistent with the recommendations of the European Association of Cardiovascular Imaging and the American Society of Echocardiography [17]. Given the retrospective design of this study, echocardiographic measurements were extracted from existing clinical reports, and data availability varied across parameters. Particular attention was paid to right heart chambers. The following parameters were collected, when available: tricuspid annular plane systolic excursion (TAPSE, mm; available in 81 patients), tricuspid annular peak systolic velocity on tissue Doppler imaging (S′ TDI, cm/s), systolic pulmonary artery pressure (PAPs, mmHg; available in 112 patients), estimated from the peak tricuspid regurgitation velocity using the simplified Bernoulli equation (PAPs = 4 × [peak TR velocity]^2^ + estimated right atrial pressure). Right atrial pressure was estimated from the diameter and inspiratory collapsibility of the inferior vena cava, in accordance with the recommendations of the American Society of Echocardiography [12]. In the absence of an adequate tricuspid regurgitation envelope, PAPs was considered non-measurable and treated as missing. The TAPSE/PAPs ratio as a non-invasive surrogate of right ventricular–pulmonary arterial (RV–PA) coupling (available in 71 patients), the peak velocity of tricuspid regurgitation (TR velocity, m/s), and the semi-quantitative grade of tricuspid regurgitation. Standard left ventricular and left atrial parameters were also recorded when available. Patients with non-measurable echocardiographic values (e.g., absent tricuspid regurgitation jet preventing reliable sPAP estimation) were considered as missing for the corresponding analyses, and all statistical analyses involving echocardiographic data were performed on the available complete-case subsets, with sample sizes reported for each analysis.

### 2.5. Ethical Considerations

This study was conducted in accordance with the principles of the Declaration of Helsinki and the guidelines of Good Clinical Practice. Given the retrospective and observational nature of this study and the use of anonymized data extracted from routine clinical practice, the requirement for formal informed consent was waived according to local regulations. All data were handled in compliance with current privacy regulations (GDPR, Regulation EU 2016/679).

### 2.6. Statistical Analysis

The normal distribution of data was checked using the Shapiro–Wilk test, and all continuous variables were found to follow normality. Continuous variables were presented as mean ± standard deviation and categorical variables were reported as absolute frequencies and percentages. One-way ANOVA or Chi-square test was applied to find the differences between quantitative parameters or qualitative parameters between groups, respectively.

Pearson’s correlation coefficient (r) was assessed to find the association between pulmonary function parameters and echocardiographic indices. To evaluate the independent association between DLCO and echocardiographic parameters of RV function, multivariable linear regression models were constructed, using TAPSE, PAPs, and TAPSE/PAPs as dependent variables. DLCO was entered into the models both as a continuous variable and as a categorical variable (with the Mild DLCO group, 60–79% predicted, used as the reference category). The data was adjusted for age, sex, and smoking status in the subset of patients in whom this variable was available. Regression coefficients (β) were reported with their 95% confidence intervals.

To compare the relative predictive value of different pulmonary function parameters across the same right ventricular outcomes, supplementary multivariable linear regression models were constructed for each predictor (DLCO-Sb, DLCO/VA, FVC, FEV1, TLC, FVC/DLCO ratio, and TLC/DLCO ratio) paired with each RV outcome (TAPSE, PAPs, and TAPSE/PAPs). To allow for direct comparison of effect sizes across predictors expressed in different units, all predictor and outcome variables were standardized to a z-score before being entered in the regression models, using the formula z = (x − $\bar{X}$)/s, where x is the individual observed value, $\bar{X}$ is the sample mean of that variable, and s is its sample standard deviation. After this transformation, every variable had a mean of 0 and a standard deviation of 1. The resulting standardized regression coefficients (β) represent the change, in standard deviations of the outcome, associated with a one–standard-deviation increase in the predictor, and were directly comparable across predictors with different scales of measurement [18]. All comparative models were adjusted for age, sex, and smoking status.

A two-sided *p*-value < 0.05 was considered statistically significant. All statistical analyses were performed using standard statistical software (IBM SPSS Statistics, version 26.0; IBM Corp., Armonk, NY, USA).

## 3. Results

### 3.1. Study Population and Baseline Characteristics

A total of 198 patients with confirmed IPF, 188 of whom completed PFT, were included in this study. They were then stratified into four severity categories based on DLCO-Sb: Normal (DLCO ≥ 80% predicted, n = 21, 11.2%), Mild (60–79%, n = 56, 29.8%), Moderate (40–59%, n = 70, 37.2%), and Severe (<40%, n = 41, 21.8%) (Table 1).

The population was predominantly male (84.6%), with a mean age of 72 ± 8 years, in line with the typical demographic profile of IPF. The distribution of sex differed significantly across DLCO categories (*p* = 0.042), with males more represented in the more severe DLCO groups. Smoking history was available for 182 patients: 23.6% were never-smokers, 71% former smokers, and 5.4% current smokers, without significant differences across DLCO categories (*p* = 0.38). Body mass index (26.4 ± 4.3 kg/m^2^) and serum vitamin D levels (mean 20.3 ng/mL) were similar across DLCO strata (*p* = 0.78 and *p* = 0.59, respectively) (Table 1). The main cardiovascular comorbidities were hypertension (56.9%), dyslipidemia (56.9%), and diabetes mellitus (23.4%), with no significant differences across DLCO categories (all *p* > 0.5) (Table 1).

### 3.2. Echocardiographic and Pulmonary Function Parameters by DLCO Severity

Across DLCO categories, the main echocardiographic parameters of the right heart showed a progressive deterioration with decreasing gas exchange capacity. TAPSE tended to be lower in patients with more severe DLCO impairment (Normal 19.95 ± 2.18 mm; Mild 20.71 ± 2.02 mm; Moderate 19.26 ± 1.79 mm; Severe 19.03 ± 3.76 mm) (*p* = 0.054) (Table 1).

PAPs showed a statistically significant difference across DLCO categories (*p* = 0.023), being lowest in the Mild group (31.67 ± 10.67 mmHg) and highest in patients with Severe DLCO reduction (45.57 ± 23.13 mmHg), supporting the hypothesis of increased pulmonary vascular load in patients with more impaired gas exchange (Table 1).

The TAPSE/PAPs ratio, a non-invasive surrogate of right ventricular–pulmonary arterial (RV–PA) coupling, was available in 71 patients (Normal, n = 10; Mild, n = 15; Moderate, n = 19; Severe, n = 27) and differed significantly across DLCO strata (one-way ANOVA, *p* = 0.040). The highest mean value was observed in the Mild group (0.68 ± 0.21 mm/mmHg), with an intermediate value in the Normal group (0.59 ± 0.18) and the lowest values in the Moderate (0.57 ± 0.23) and Severe (0.56 ± 0.44) groups. The pattern across the four ordered categories was therefore not strictly monotonic: rather than declining stepwise from Normal to Severe, the ratio peaked in the Mild category and was lower in the Moderate and Severe categories. The comparatively low mean in the Normal group should be interpreted with caution, given the small size and clinical heterogeneity of this subgroup and the wide dispersion of values (particularly in the Severe group, SD 0.44) (Table 1).

As expected, pulmonary function parameters differed markedly across DLCO strata: DLCO-Sb, DLCO/VA, FVC, FEV1, TLC, FVC/DLCO, and TLC/DLCO (*p* < 0.001), all showed progressive and highly significant reductions with increasing disease severity (Table 1).

Antifibrotic therapy had been prescribed in 180 patients (90.9%), while 18 (9.1%) had no antifibrotic drug recorded. The most frequently used first-line agent was nintedanib (112 patients, 56.6%), followed by pirfenidone (68 patients, 34.3%). During follow-up, 15 patients (7.6%) were switched to the alternative antifibrotic agent (9 from pirfenidone to nintedanib and 6 from nintedanib to pirfenidone). The distribution of antifibrotic treatment did not differ significantly across DLCO severity categories (*p* = 0.43) (Table 1).

The HRCT pattern at diagnosis was documented in 165 patients (83.3%). Among these, a definite UIP pattern was the most frequent (76 patients, 46.1%), followed by probable UIP (63 patients, 38.2%) and an indeterminate-for-UIP pattern (26 patients, 15.8%); no scan was classified as an alternative diagnosis, consistent with the confirmed diagnosis of IPF. The distribution of HRCT patterns did not differ significantly across DLCO severity categories (*p* = 0.108) (Table 1).

### 3.3. Correlation Between DLCO and Echocardiographic Parameters

**Figure 1:**
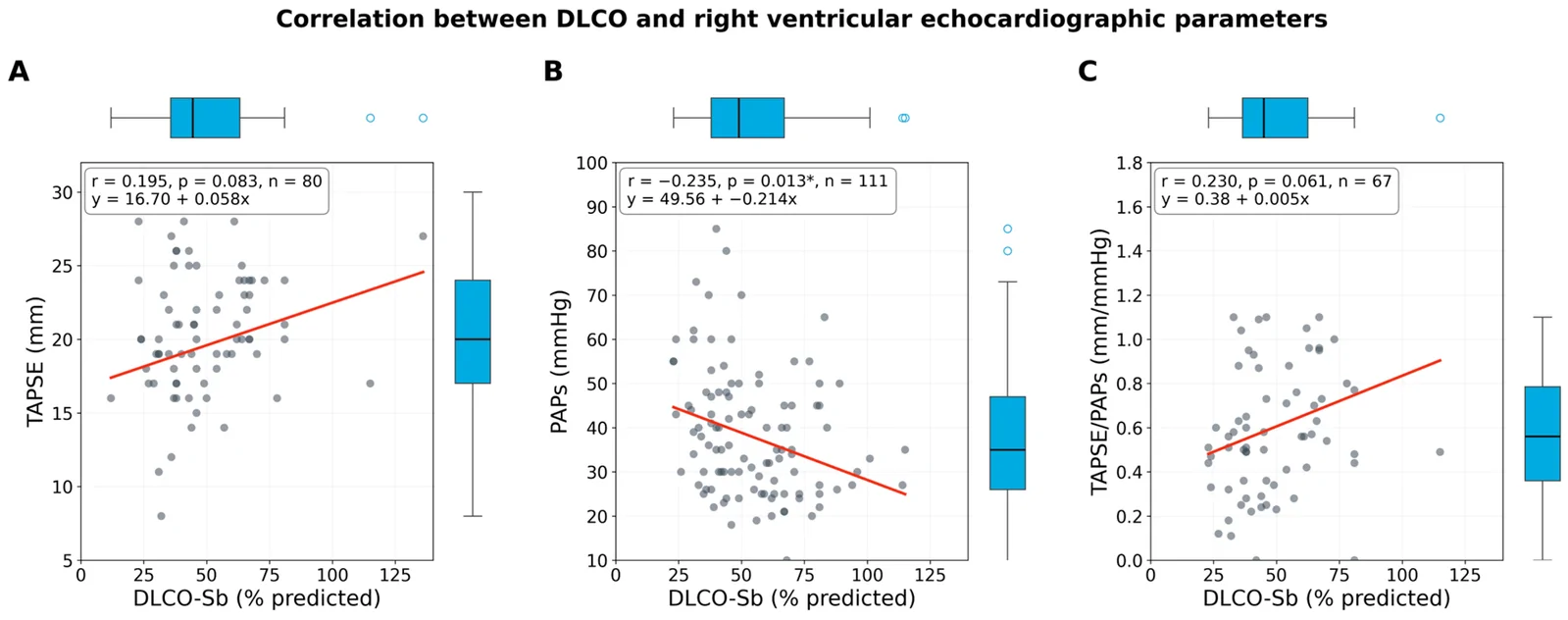
Scatter plots with marginal box plots illustrating the relationship between DLCO and echocardiographic parameters of right ventricular function: (**A**) TAPSE (mm), (**B**) PAPs (mmHg), and (**C**) the TAPSE/PAPs ratio (mm/mmHg). In each panel, every point represents an individual patient, and the red line is the least-squares linear regression fit; the inset reports the Pearson correlation coefficient (r), its *p*-value (* *p* < 0.05), the number of patients with paired data (n), and the regression equation. The marginal box plots show the distribution of each variable—DLCO-Sb (% predicted) along the horizontal axis (top) and the corresponding echocardiographic parameter along the vertical axis (right). In each box plot, the central line indicates the median, the box spans the interquartile range (IQR; 25th to 75th percentiles), and the whiskers extend to the most extreme values within 1.5 × IQR of the box; open circles beyond the whiskers denote outliers. DLCO: diffusing capacity of the lung for carbon monoxide; TAPSE: tricuspid annular plane systolic excursion; PAPs: systolic pulmonary artery pressure.

Correlation analyses were performed to assess the relationships between DLCO and the main echocardiographic indices of right ventricular function (Figure 1). A weak positive correlation was observed between DLCO and TAPSE (r = 0.195, *p* = 0.083), suggesting a trend toward better right ventricular longitudinal systolic function with higher DLCO values; however, this association did not reach statistical significance. In contrast, DLCO showed a weak but statistically significant inverse correlation with PAPs (r = −0.235, *p* = 0.013), indicating that lower DLCO values were associated with higher pulmonary artery pressures and, consequently, greater pulmonary vascular load. A weak but statistically significant positive correlation was also observed between DLCO and the TAPSE/PAPs ratio (r = 0.248, *p* = 0.041), suggesting that better pulmonary gas exchange was associated with more preserved right ventricular–pulmonary arterial coupling. Overall, although the correlations between DLCO and PAPs and between DLCO and the TAPSE/PAPs ratio were weak, their statistical significance suggests a meaningful association between impaired pulmonary diffusion capacity, increased pulmonary vascular load, and reduced right ventricular–pulmonary arterial coupling.

### 3.4. Regression Analysis

**Figure 2:**
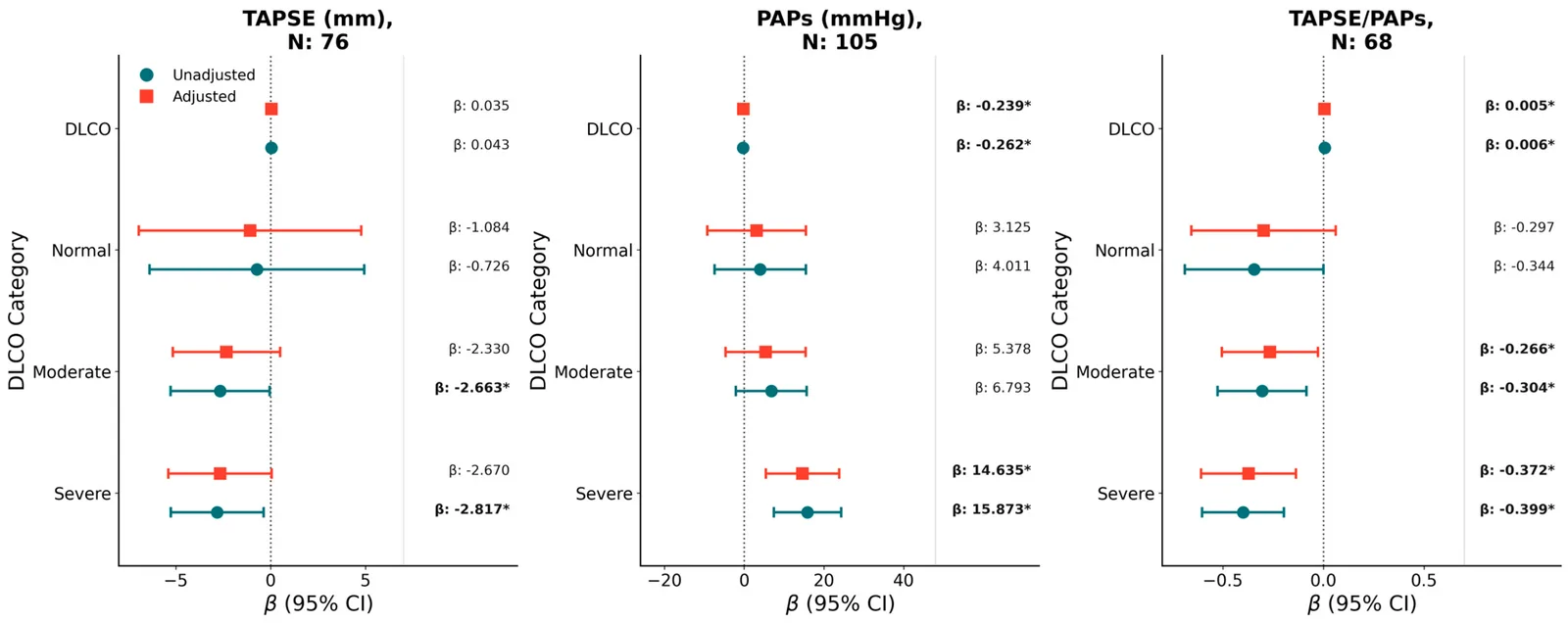
Univariable and multivariable linear regression models evaluating the independent association between DLCO and echocardiographic indices of right ventricular function TAPSE (mm), PAPs (mmHg), and TAPSE/PAPs ratio. DLCO: Diffusing Capacity for Carbon Monoxide; TAPSE: Tricuspid Annular Plane Systolic Excursion; PAPs: Pulmonary Artery Pressure systolic. DLCO was categorized as normal, mild, moderate, or severe impairment, with the mild category used as the reference group. Bold text and asterisks indicate statistically significant findings.

To further explore the association between DLCO and right ventricular function, multivariable linear regression analyses were performed, using TAPSE, PAPs, and TAPSE/PAPs as dependent variables. DLCO was evaluated both as a continuous variable and as a categorical variable. For the categorical analyses, the Mild DLCO group (60–79%) was chosen as the reference category, as it represented the largest subgroup with complete echocardiographic data and provided the most stable reference estimates. Each model was tested in an unadjusted version and in a version adjusted for age, sex, and smoking status (Figure 2).

Based on results presented in Figure 2, higher DLCO values were significantly associated with lower PAPs, both in the unadjusted model (β = −0.262, 95% CI: −0.417 to −0.108, *p* = 0.001) and after adjustment for age, sex, and smoking (β = −0.239, 95% CI: −0.398 to −0.080, *p* = 0.004), indicating that each 1% increase in DLCO corresponds to an approximate 0.24 mmHg decrease in PAPs.

DLCO was also positively associated with the TAPSE/PAPs ratio in both the unadjusted model (β = 0.0061, 95% CI: 0.0015 to 0.0108, *p* = 0.011) and after adjustment (β = 0.0052, 95% CI: 0.0003 to 0.0100, *p* = 0.040), indicating that higher DLCO values are associated with better RV–PA coupling independently of demographic and smoking variables. For TAPSE, a positive trend was observed (unadjusted β = 0.043, *p* = 0.075; adjusted β = 0.035, *p* = 0.155), but the association did not reach statistical significance.

When DLCO was analyzed categorically, patients with severe DLCO reduction (<40%) showed significantly higher PAPs compared with the mild reference group, both in the unadjusted model (β = 15.87 mmHg, 95% CI: 7.43 to 24.31, *p* < 0.001) and after adjustment for age, sex, and smoking (β = 14.63 mmHg, 95% CI: 5.43 to 23.84, *p* = 0.002). The moderate DLCO group showed a positive but non-significant increase in PAPs compared with the Mild group (β = 5.378, 95% CI: −1.938 to 11.89, *p* = 0.15) (Figure 2).

The TAPSE/PAPs ratio was significantly lower in both the severe and moderate DLCO groups compared with the mild reference. In the unadjusted model, patients with severe DLCO impairment showed a marked reduction in TAPSE/PAPs (β = −0.399, 95% CI: −0.603 to −0.196, *p* < 0.001), as did patients with moderate impairment (β = −0.304, 95% CI: −0.525 to −0.084, *p* = 0.008). After adjustment for age, sex, and smoking, these associations remained statistically significant (Severe: β = −0.372, *p* = 0.002; Moderate: β = −0.266, *p* = 0.029), indicating an independent graded association between worsening DLCO and progressive deterioration of RV–PA coupling (Figure 2).

Regarding TAPSE, patients with severe and moderate DLCO impairment showed lower values than the mild reference group in the unadjusted analysis (Severe: β = −2.82 mm, *p* = 0.024; Moderate: β = −2.66 mm, *p* = 0.046). After adjustment, these associations showed a consistent trend in the same direction, with borderline statistical significance for the severe category (β = −2.67 mm, *p* = 0.055) and loss of significance for the moderate category (β = −2.33 mm, *p* = 0.106) (Figure 2).

The Normal DLCO group (≥80%) did not differ significantly from the mild reference group in any of the models (for TAPSE/PAPs: β = −0.297, *p* = 0.13; for PAPs: β = 3.125, *p* = 0.33), likely reflecting the limited sample size and clinical heterogeneity of this subgroup (Figure 2).

### 3.5. Comparison of DLCO with Other Pulmonary Function Parameters

**Figure 3:**
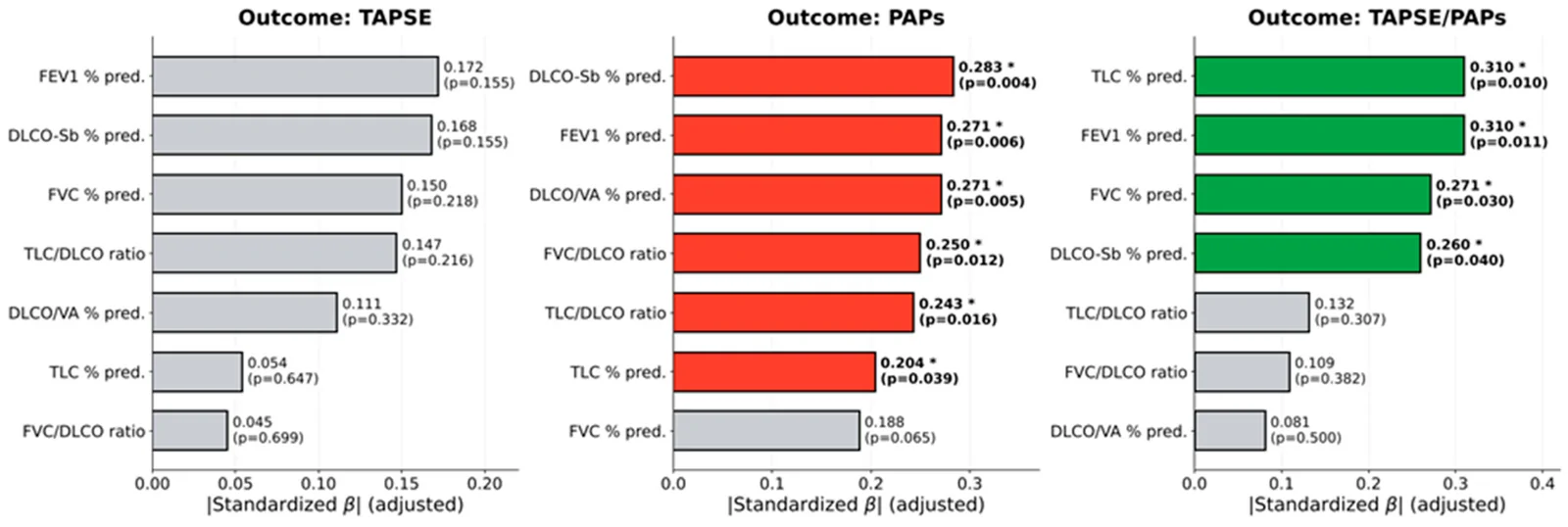
Comparative analysis of pulmonary function parameters as predictors of right ventricular echocardiographic indices in patients with idiopathic pulmonary fibrosis. FVC: Forced Vital Capacity, FEV1: Forced Expiratory Volume in 1 s, TLC: Total Lung Capacity, DLCO: Diffusing Capacity for Carbon Monoxide; TAPSE: Tricuspid Annular Plane Systolic Excursion; PAPs: Pulmonary Artery Pressure systolic. Gray: non-significant association (*p* ≥ 0.05). Red: significant association with PAPs, an adverse hemodynamic marker (*p* < 0.05). Green: significant association with TAPSE/PAPs, a favorable RV–PA coupling index (*p* < 0.05). Asterisks denote *p* < 0.05. Bold text and asterisks indicate statistically significant findings.

To assess the relative predictive value of DLCO compared with other commonly available pulmonary function parameters, we performed a supplementary multivariable regression analysis including FVC, FEV1, TLC, DLCO/VA, FVC/DLCO, and TLC/DLCO ratios. To allow for direct comparison across variables with different units, all predictors and outcomes were standardized (Z-scored), and standardized β coefficients (Std β) were calculated for each predictor–outcome pair, adjusted for age, sex, and smoking status (Figure 3).

For PAPs, DLCO emerged as the strongest predictor (Std β = −0.283, *p* = 0.004), followed by DLCO/VA (Std β = −0.271, *p* = 0.005) and FEV1 (Std β = −0.271, *p* = 0.006). The composite ratios FVC/DLCO and TLC/DLCO also showed significant but slightly weaker associations (Std β = +0.250, *p* = 0.012 and Std β = +0.243, *p* = 0.016, respectively). TLC reached statistical significance (Std β = −0.204, *p* = 0.039), while FVC showed a non-significant trend (Std β = −0.188, *p* = 0.065).

For the TAPSE/PAPs ratio, FEV1 and TLC emerged as the strongest predictors (Std β = +0.310, *p* = 0.011 and Std β = +0.310, *p* = 0.010, respectively), followed by FVC (Std β = +0.271, *p* = 0.030) and DLCO (Std β = +0.260, *p* = 0.040). The FVC/DLCO, TLC/DLCO ratios and DLCO/VA were not significantly associated with the TAPSE/PAPs ratio in the adjusted models.

For TAPSE, none of the PFT parameters reached statistical significance after adjustment, although a consistent trend was observed across all predictors, with FEV1 showing the largest absolute effect size (Std β = +0.172, *p* = 0.155).

These findings confirm that DLCO showed the strongest association with pulmonary artery pressure and, indicate that while DLCO reflects the principal component of pulmonary vascular involvement, other PFT parameters particularly TLC and FEV1 may also contribute to the prediction of RV–PA coupling.

## 4. Discussion

The present study evaluated the association between pulmonary gas exchange, as assessed by the single-breath diffusing capacity for carbon monoxide (DLCO-Sb), and echocardiographic indices of right ventricular (RV) function in a cohort of patients with idiopathic pulmonary fibrosis (IPF). Three principal findings emerged from the analysis. First, a progressive and statistically significant increase in PAPs and a lower TAPSE/PAPs ratio in patients with moderate-to-severe versus mild DLCO impairment. Second, DLCO, analyzed both as a continuous variable and as a categorical variable using the Mild impairment group (60–79% predicted) as the reference, was independently associated with PAPs and with RV–pulmonary arterial (RV–PA) coupling, even after adjustment for age, sex, and smoking status. Third, the association between DLCO and TAPSE showed a consistent trend but did not reach statistical significance in most models, suggesting that intrinsic RV contractility is preserved in the majority of IPF patients, while the afterload and the RV–PA coupling are the first to deteriorate.

Our finding of an inverse association between DLCO and PAPs is consistent with a large body of evidence linking reduced diffusing capacity to pulmonary vascular involvement in IPF. Pulmonary hypertension is a well-recognized complication of IPF, with a prevalence that increases with disease severity and is associated with a markedly worse prognosis [19]. Previous studies have shown that a disproportionate reduction in DLCO, out of proportion to the reduction in lung volumes, is a relatively early indicator of pulmonary vascular remodeling and of the development of PH (pulmonary hypertension) in interstitial lung diseases [20,21]. Zisman and colleagues demonstrated that a reduced DLCO, especially when combined with the need for supplemental oxygen or with a high FVC/DLCO ratio, improves the non-invasive prediction of PH in IPF [22,23], and similar conclusions have been drawn from several subsequent cohorts [24,25]. Our data extend these observations by quantifying the dose–response relationship between DLCO and PAPs: each 1% decrease in DLCO corresponded to an approximate 0.24 mmHg increase in PAPs after adjustment for confounders, and patients with severe DLCO impairment (<40%) showed, on average, a 14.6 mmHg higher PAPs compared with those with only mild impairment.

The pathophysiological basis for this association is likely multifactorial. The destruction of the alveolar–capillary interface caused by progressive fibrotic remodeling reduces the available surface area for gas exchange, while concomitant vascular rarefaction, intimal fibrosis, and medial hypertrophy of the pulmonary arterioles increase pulmonary vascular resistance [16,26,27]. DLCO therefore integrates information on both the alveolar–capillary membrane and the pulmonary microvasculature, making it a particularly sensitive functional marker of pulmonary vascular burden in IPF [5].

One of the most relevant findings of the present study is the consistent and independent association between DLCO and the TAPSE/PAPs ratio. This parameter, originally proposed by Guazzi et al., has been extensively validated as a non-invasive surrogate of the invasively measured ratio of RV end-systolic elastance to pulmonary arterial elastance (Ees/Ea] and is now widely accepted as a reliable marker of RV–PA coupling [10]. A TAPSE/PAPs ratio below 0.31 mm/mmHg has been shown to independently predict mortality in patients with severe pulmonary arterial hypertension, and its prognostic role has been subsequently confirmed in heart failure, systemic sclerosis, and more recently in fibrotic interstitial lung diseases [11,28]. In systemic sclerosis complicated by pulmonary hypertension, a reduced TAPSE/sPAP ratio has also been associated with cardiovascular events and mortality, further supporting its clinical value as a marker of RV–PA uncoupling across different pulmonary vascular phenotypes [25]. Santoro and colleagues specifically demonstrated that reduced TAPSE/PAPs identifies fibrotic ILD patients at higher risk of adverse outcomes, and proposed this parameter as a screening tool for early cardiopulmonary involvement [29,30].

In our cohort, patients with severe DLCO reduction showed a markedly lower TAPSE/PAPs ratio compared with the Mild reference group (adjusted β = −0.37, *p* = 0.002), and a similar, though smaller, effect was observed in the Moderate group (adjusted β = −0.27, *p* = 0.029). The graded nature of this association across DLCO strata and its persistence after multivariable adjustment support the hypothesis that DLCO and TAPSE/PAPs capture complementary aspects of the progressive cardiopulmonary deterioration in IPF: DLCO reflects the progressive loss of the alveolar–capillary unit, while TAPSE/PAPs is associated with the functional consequence of the increased afterload on the right ventricle [16,31]. The combined use of these two parameters may therefore offer a more complete, integrated assessment of cardiopulmonary involvement than either parameter alone, and may help to identify a subgroup of IPF patients at higher risk of RV failure and mortality [29,32].

In contrast to the significant associations observed for PAPs and the TAPSE/PAPs ratio, the association between DLCO and TAPSE alone was weaker and did not reach statistical significance in the fully adjusted models. This is biologically plausible and consistent with previous reports indicating that TAPSE may remain within normal limits despite right ventricular pressure overload, as the right ventricle can initially compensate through increased contractility [32,33]. In this setting, subclinical right ventricular dysfunction is more sensitively detected by parameters such as right ventricular longitudinal strain, three-dimensional ejection fraction, or composite indices of RV–PA interaction [34,35]. D’Andrea and colleagues reported that IPF patients with preserved TAPSE may nonetheless show abnormalities in speckle-tracking strain and exercise reserve, and other studies have found the TAPSE/PAPs ratio to be a more sensitive marker of RV–PA uncoupling than any individual parameter [36]. Consistent with this, in our cohort TAPSE remained within normal limits even among patients with marked DLCO impairment, whereas the TAPSE/PAPs ratio was already reduced, reflecting a mismatch between right ventricular contractility and afterload [37].

To put our findings into a broader functional context, we also compared DLCO with other commonly available pulmonary function parameters FVC, FEV1, TLC, DLCO/VA, FVC/DLCO ratio, and TLC/DLCO ratio) in a supplementary analysis using standardized regression coefficients. This approach allowed us to rank the relative strength of each predictor against the same set of right ventricular outcomes [18]. DLCO showed the strongest association with systolic pulmonary artery pressure PAPs), supporting its central role as a non-invasive marker of pulmonary vascular involvement in IPF [20,22]. The pattern observed for the TAPSE/PAPs ratio was different from that for PAPs: while DLCO remained significantly associated with RV–PA coupling, TLC and FEV1 emerged as comparably strong—or even marginally stronger—predictors. This is biologically plausible when the composite nature of the TAPSE/PAPs ratio is considered. Unlike PAPs, which reflects afterload alone and is therefore most closely tied to the pulmonary vascular compartment that DLCO interrogates, the TAPSE/PAPs ratio integrates both right ventricular afterload and contractility, and thus responds to the total cardiopulmonary burden of the disease rather than to vascular remodeling in isolation. In IPF, that burden is not purely vascular: progressive fibrosis reduces total lung capacity and, through parenchymal retraction, mechanically distorts and compresses the alveolar capillary bed, promotes hypoxic pulmonary vasoconstriction, and increases pulmonary vascular resistance [5,15,16]. Reduced lung volumes also lower thoracic and lung compliance, which, together with ventricular interdependence within the constrained thorax, can impair right ventricular filling and longitudinal excursion [33,35]. TLC therefore serves as a global marker of fibrotic severity that captures this combined mechanical and vascular loading, while FEV1 in this restrictive population behaves largely as a further volume-dependent index rather than a marker of airflow obstruction, and consequently co-varies with overall disease severity [13]. Because all of these pulmonary function parameters decline together as the disease advances, they carry substantially overlapping information, and the TAPSE/PAPs ratio—being a composite index—appears to be driven by this integrated mechanical–vascular burden rather than by any single functional compartment [29,38]. It should be emphasized, however, that the standardized regression coefficients for DLCO, TLC, and FEV1 were of similar magnitude with overlapping confidence intervals; the apparent equivalence therefore reflects comparable, partly redundant associations rather than a demonstrable superiority of one parameter over another, and the analysis was not powered to distinguish small differences between correlated predictors [36,38,39]. In contrast, none of the PFT parameters showed a significant independent association with TAPSE alone after adjustment, reinforcing the previously discussed concept that conventional measures of RV longitudinal systolic function may remain preserved while afterload-related abnormalities and the associated uncoupling are already detectable. The composite ratios FVC/DLCO and TLC/DLCO, although significantly associated with PAPs, did not outperform DLCO alone in our cohort, and showed weaker associations with the TAPSE/PAPs ratio, suggesting that at least in this IPF population these composite indices do not provide substantial added value over DLCO for the assessment of RV–PA coupling.

The findings of the present study may have potential clinical relevance, although they should be interpreted with caution given the retrospective, cross-sectional design. They are consistent with the view that DLCO, a simple and widely available functional marker, carries information beyond gas exchange that may relate indirectly to the pulmonary vasculature and the right ventricle. Our data also raise the hypothesis that combining DLCO with an echocardiographic index of RV–PA coupling, such as the TAPSE/PAPs ratio, could help characterize the burden of right heart involvement in IPF. Whether this combination has value as a screening or risk-stratification tool for example, to identify patients who might benefit from closer cardiopulmonary evaluation, from referral for dedicated pulmonary hypertension assessment, or from therapies approved for PH associated with interstitial lung disease [40] cannot be established from the present analysis and would need to be tested prospectively against right heart catheterization and clinical outcomes. Our results should therefore be regarded as generating hypotheses for such future studies rather than as a basis for changes in current clinical practice.

The present study has several strengths, including a relatively large, well-characterized IPF cohort, standardized pulmonary function and echocardiographic protocols, and a statistical approach combining correlation, stratified comparison, and multivariable regression. Several limitations should nonetheless be acknowledged. First, the retrospective, single-center design carries a risk of selection bias and limits generalizability. Second, echocardiographic data were incomplete: the TAPSE/PAPs ratio was available in only 71 patients (35.9%); so, analyses of this parameter were performed on a complete-case subset with reduced power and wider confidence intervals and should be regarded as exploratory and hypothesis-generating. Third, pulmonary pressures were estimated echocardiographically rather than by right heart catheterization, and may be over- or under-estimated, particularly with suboptimal tricuspid regurgitation envelopes. Fourth, the cross-sectional design precludes conclusions about temporal sequence, causality, or prognosis; the findings represent associations at a single time point and require confirmation in prospective studies with clinical outcomes. Fifth, concomitant emphysema (CPFE) was recorded in only a minority of patients (present in 11, absent in 25); as CPFE increases the risk of pulmonary hypertension and independently lowers DLCO, unmeasured emphysema is a potential residual confounder warranting dedicated study. Larger, prospective, multicenter studies incorporating right heart catheterization and standardized HRCT assessment are needed to confirm and extend these findings.

## 5. Conclusions

In this retrospective single-center study of patients with idiopathic pulmonary fibrosis, lower DLCO was associated with higher echocardiographic PAPs and a lower TAPSE/PAPs ratio. In an exploratory comparison of pulmonary function parameters, DLCO showed the strongest association with PAPs, while other parameters of lung volumes and airflow were also related to indices of RV–pulmonary arterial coupling. TAPSE alone was not independently associated with DLCO after adjustment, which is consistent with previous observations that conventional measures of RV longitudinal function may remain within the normal range in earlier stages of pulmonary vascular involvement.

These findings are cross-sectional and associative and should be regarded as hypothesis-generating. They indicate that, in this IPF cohort, more severely impaired gas exchange was associated with higher estimated pulmonary pressures and reduced RV–PA coupling, but they do not establish causality, temporal sequence, or prognostic value, and are not intended to guide pulmonary hypertension screening or treatment decisions. Whether the combined evaluation of DLCO and the TAPSE/PAPs ratio has clinical utility for characterizing or stratifying right heart involvement in IPF will need to be determined in prospective studies with complete echocardiographic ascertainment, right heart catheterization, and long-term clinical outcomes.

## Figures and Tables

**Table 1:** Baseline demographic, clinical, and functional characteristics of patients with idiopathic pulmonary fibrosis stratified into four DLCO severity groups.

		DLCO (188)				
		Normal (≥80)	Mild (60–79)	Moderate (40–59)	Severe (<40)	*p*-Value
Number, %		21 (11.2%)	56 (29.8%)	70 (37.2)	41 (21.8)	
Age, y		68.4 ± 9.3	71.56 ± 8.75	73.48 ± 7.64	72.48 ± 7.94	0.087
Sex	Male (159)	19 (11.9)	40 (25.2)	62 (39)	38 (23.9)	0.042
	Female (29)	2 (6.9)	16 (55.2)	8 (27.6)	3 (10.3)	
Smoking (182)	No (43)	7 (16.3)	13 (30.2)	15 (34.9)	8 (18.6)	0.38
	Ex (129)	14 (10.9)	37 (28.7)	47 (36.4)	31 (24)	
	Current (10)	0	2 (20)	7 (70)	1 (10)	
BMI, Kg/m^2^		26.4 ± 4.32	26.69 ± 3.07	26.72 ± 5.12	25.59 ± 3.72	0.78
VitD, ng/mL		25.7 ± 6.62	18.8 ± 11.27	18.68 ± 10.11	20.58 ± 14.74	0.59
Comorbidities						
Diabetes (44)		4 (19)	16 (30.8)	14 (20.3)	10 (25)	0.54
Hypertension (107)		12 (57.1)	29 (55.8)	41 (60.3)	25 (62.5)	0.91
Dyslipidemia (107)		14 (66.7)	29 (55.8)	41 (59.4)	23 (57.5)	0.85
Echocardiography						
TAPSE, mm (n: 85)		19.95 ± 2.18	20.71 ± 2.02	19.26 ± 1.79	19.03 ± 3.76	0.054
PAPs, mmHg (n: 121)		36.47 ± 12.28	31.67 ± 10.67 ^a^	36.98 ± 18.53 ^b^	45.57 ± 23.13 ^b^	0.023
TAPSE/PAPs (n: 71)		0.59 ± 0.18	0.68 ± 0.21^a^	0.57 ± 0.23^b^	0.56 ± 0.44 ^b^	0.04
Respiratory						
FVC, %		96.65 ± 16.21	96.33 ± 19.82	89.20 ± 16.33 ^ab^	78.53 ± 22.46 ^abc^	<0.001
FEV1, %		106.05 ± 16.51	102.67 ± 19.02	94.79 ± 17.39 ^ab^	83.22 ± 17.61 ^abc^	<0.001
TLC %		86.63 ± 12.07	90.29 ± 18.55	86.06 ± 14.37	77.67 ± 16.85 ^abc^	0.001
Antifibrotic therapy						
Nintedanib (107)		8 (7.5)	32 (29.9)	41 (38.3)	26 (24.3)	0.43
Pirfenidone (65)		9 (13.8)	21 (32.3)	24 (36.9)	11 (16.9)	0.41
None/not recorded (15)		4 (26.7)	2 (13.3)	5 (33.3)	4 (26.7)	
HRCT pattern						
Definite UIP (73)		3 (4.1)	17 (23.3)	28 (38.4)	25 (34.2)	0.11
Probable UIP (59)		5 (8.5)	20 (33.9)	24 (40.7)	10 (16.9)	0.15
Indeterminate for UIP (24)		3 (12.5)	10 (41.7)	8 (33.3)	3 (12.5)	0.1

Data presented as mean and standard division or number and percentage. Chi-square test or one-way ANOVA has been done. Tukey post hoc analysis: ^a^: normal vs. mild, moderate and severe, ^b^: mild vs. moderate and severe, ^c^: moderate vs. severe. BMI: Body Mass Index; VitD: Vitamin D; FVC: Forced Vital Capacity, FEV1: Forced Expiratory Volume in 1 s, TLC: Total Lung Capacity, DLCO: Diffusing Capacity for Carbon Monoxide; TAPSE: Tricuspid Annular Plane Systolic Excursion; PAPs: Pulmonary Artery Pressure systolic. Percentages and counts refer to the 188 patients with an available DLCO measurement; 3 patients with no recorded antifibrotic therapy lacked DLCO and are not shown. Fifteen patients switched to the alternative antifibrotic agent during follow-up and are classified here by their first-line drug. HRCT pattern available in 165 patients; percentages refer to the DLCO-stratified subset.

## Data Availability

The datasets used and/or analysed during the current study are available from the corresponding author on reasonable request.

## Abbreviations

The following abbreviations are used in this manuscript:

ALAT	Latin American Thoracic Association
ANOVA	analysis of variance
ASE	American Society of Echocardiography
ATS	American Thoracic Society
BMI	body mass index
CI	confidence interval
DLCO	Diffusing Capacity for Carbon Monoxide
EACVI	European Association of Cardiovascular Imaging
Ees/Ea	right ventricular end-systolic elastance/pulmonary arterial elastance
ERS	European Respiratory Society
ESC	European Society of Cardiology
FVC	Forced Vital Capacity
FEV1	Forced Expiratory Volume in 1 s
GDPR	General Data Protection Regulation
HRCT	high-resolution computed tomography
ILD	interstitial lung disease
IPF	idiopathic pulmonary fibrosis
JRS	Japanese Respiratory Society
MCHC	mean corpuscular hemoglobin concentration
MCV	mean corpuscular volume
PAPs	Pulmonary Artery Pressure systolic
PFT	pulmonary function test
PH	pulmonary hypertension
PTH	parathyroid hormone
RDW	red cell distribution width
RV	right ventricular
RV–PA	RV–pulmonary arterial
SD	standard deviation
S′ TDI	tissue Doppler-derived systolic velocity
TAPSE	Tricuspid Annular Plane Systolic Excursion
TLC	Total Lung Capacity
TR	tricuspid regurgitation
UIP	usual interstitial pneumonia
